# Preparation and Properties of Crystalline IGZO Thin Films

**DOI:** 10.3390/membranes11020134

**Published:** 2021-02-14

**Authors:** Xiao Wang, Zhihua Shen, Jie Li, Shengli Wu

**Affiliations:** 1 School of Electronic Information and Artificial Intelligence, Shaanxi University of Science and Technology, Xi’an 710049, China; 2 School of Electronics and Information Engineering, Nantong Vocational University, Nantong 226007, China; 9000082@mail.ntvu.edu.cn; 3 Key Laboratory for Physical Electronics and Devices of the Ministry of Education, School of Electronic Science and Engineering, Xi’an Jiaotong University, Xi’an 710049, China; jie_li@mail.xjtu.edu.cn

**Keywords:** indium gallium zinc oxide (IGZO) thin film, RF sputtering, c-axis aligned crystal IGZO, corrosion resistance

## Abstract

IGZO thin films can be used as active layers of thin-film transistors and have been widely studied. However, amorphous indium gallium zinc oxide (IGZO) fabricated at room temperature is vulnerable in subsequent manufacturing processes, such as etching and sputtering; this limits IGZO thin film transistors’ (TFTs) use in commercial products. In this paper, we prepared a c-axis crystallized IGZO thin film by Radio Frequency (RF) sputtering at 180 °C, with a 50% O_2_ ratio and 110 W power. XRD images show that the crystallized film has an obvious diffraction peak near 31°, and the spacing between the crystal surfaces was calculated to be ≈0.29 nm. The HRTEM map confirmed the above results. The stability of IGZO thin films was investigated by etching them with an acid solution. The crystalline IGZO films exhibited better acid corrosion resistance, and their anticorrosion performance was 74% higher than that of amorphous IGZO (a-IGZO) films, indicating the crystalline IGZO film can provide more stable performance in applications.

## 1. Introduction

For their particular properties, transparent-oxide thin films can be used widely in thin film transistors (TFTs) as control units in liquid crystal displays (LCDs), flexible active-matrix organic light-emitting diode (AM-OLED) displays, wearable devices, photoelectric devices, thermoelectric generators and chemical and biological sensors [1,2,3,4,5,6,7]. Among many transparent-oxide thin film semiconductor materials, the indium gallium zinc oxide (IGZO) membrane is considered the most promising active layer substitution for the traditional hydrogenated amorphous silicon (a-Si)-based TFT and the low temperature poly-silicon (LTPS)-based TFT used in the backplanes of LCD and AM-OLED displays. 

IGZO TFTs have the advantages of high mobility, a reasonable on/off ratio, high optical transparency in the visible region and a large area of deposition at low temperatures [8,9]. Although high-definition displays and foldable displays using IGZO TFTs have been made [10,11], the property stability and mass production techniques are still the main obstacles to IGZO TFTs replacing Si-based TFTs commercially. Meanwhile, in the development of large, high frequency, high resolution and low power flat panel displays for the next generation, the signal delay and aperture ratio of the gate lines and data lines are main constraints. Therefore, back-channel etched (BCE) structural TFTs are widely used for their simpler structure and fewer processes, which reduce the production costs while allowing a shorter channel length and smaller parasitic capacitance. The signal distortion could be decreased by using the BCE structure. However, the amorphous IGZO (a-IGZO) fabricated at room temperature is easily damaged by subsequent fabrication process, such as etching and sputtering, restricting the applications of IGZO TFTs in commercial products. Specifically, in commercial production and manufacturing, problems caused by damage of the back channel of IGZO TFTs are inevitable, and improvement of the dependability and elimination of the undulations in characteristics are difficult, and it seems that the achievement of massive use of IGZO TFTs could take a long time [12]. 

In order to solve the performance stability problem caused by a-IGZO in the manufacturing process, Yamazaki et al. formed a partially nanocrystalline state IGZO film in which the c-axis of the crystal is aligned, and this structure has been referred to as c-axis aligned crystal (CAAC) IGZO [13]. Devices fabricated with CAAC IGZO film exhibit similar uniformity to a-IGZO devices while improving stability and exhibiting extremely low leakage currents. Park et al. formed CAAC IGZO at above 800 °C for annealing and investigated the effect of crystalline IGZO thin films on device performance, and evaluated the device reliability of CAAC IGZO under positive/negative bias stress with/without illumination. Remarkable improvement of the device reliability for CAAC IGZO TFTs was observed, especially under the bias stress with illumination, and they believed that this came from lower defect density compared with the a-IGZO film [14]. Hiramatsu et al. proposed that the high crystallinity of CAAC IGZO is important for the inhibition of oxygen vacancies, which play an important role in the characteristics of IGZO-based devices [15]. These features allow CAAC IGZO to be used as an ideal candidate for applications in traditional displays, and it is potentially useful for various other products, such as low-power displays, non-volatile memory devices, novel sensors and large-scale integrations [16,17].

In this paper, under the premise of changing the preparation parameters of a-IGZO as little as possible, the effects of RF sputtering parameters, such as Ar/O_2_ ratio, power and temperature on the preparation of crystal IGZO thin films is discussed in detail by using the design and analysis of experiment (DOE) method. Film deposition under optimized conditions shows a high degree of crystallinity and strong c-axis alignment. Highly oriented films were obtained directly by RF sputtering at an elevated substrate temperature of 180 °C with 50% O_2_ fractions. The structure of the prepared IGZO film was characterized by X-ray diffraction (XRD) and high-resolution transmission electron microscopy (HRTEM) imaging. The corrosion resistance of IGZO films under different preparation conditions was also characterized by the etching rate. 

## 2. Materials and Methods

The alkaline-free glass substrate was gradually cleaned with acetone, alcohol and deionized water in the ultrasonic cleaning machine and used as a coating substrate directly. The IGZO film was fabricated by RF magnetron sputtering (ACS-4000-C4 magnetron sputtering instrument: ULVAC Company, Chigasaki, Japan) with different parameters followed by annealing at 400 °C. The atomic composition ratio of the sputter target was: Ga:Zn:O = 1:1:1:4. According to the preliminary investigation, the temperature, Ar/O_2_ ratio and RF power were selected as the main factors affecting the crystallization of IGZO thin films. Based on the technical specifications of the machine available, the levels were as follows: temperature from 100 to 180 °C, and it remained unchanged during preparation, Ar/O_2_ ratio from 30:30 to 30:3, power from 80 to 140 W. See Table 1.

In order to obtain the relationship between sputtering parameters and film properties as well as possible, nine experiments with three factors and three levels were carried out by DOE method. The detailed preparation parameters were as in Table 2. More specifically, in terms of temperature factor, level 1 was 100 °C; level 2 was 140 °C; level 3 was 180 °C. Similarly, in the case of the Ar/O_2_ ratio, level 1 was 30:3; level 2 was 30:15; level 3 was 30:30. For sputtering power, level 1 was 80 W; level 2 was 110 W; level 3 was 140 W. In the subsequent paragraphs, we have abbreviated conditions 1–9 as C1–C9, and the samples prepared from those conditional parameters were recorded as S1-1, S2-1 and so on.

After the film was prepared, the thickness of the membrane was measured by ellipsometer, and the influences of parameters on the deposition rate (D.R.) were investigated. The structure of prepared IGZO film was characterized by XRD (Rigaku company, Osaka, Japan) and HRTEM imaging (FEI company, Portland, OR, USA). The corrosion resistance of IGZO films under different preparation conditions was characterized by the etching rate.

## 3. Results and Discussion

### 3.1. The Effects of Sputtering Parameters on the Deposition Rate

**Figure 1:**
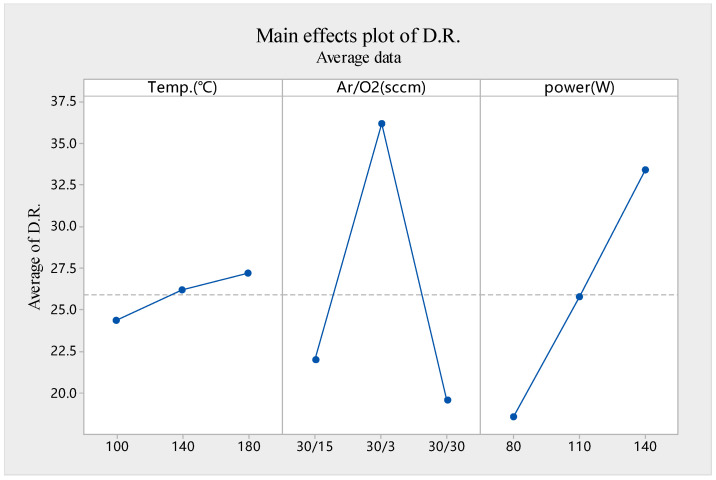
A main effects plot of the deposition rate.

In order to maintain the same thickness for subsequent XRD and HRTEM tests, we first measured the film deposition rates of different conditions. The deposition rate was obtained by the ratio of thickness to time of the thin film deposited on the glass substrate, under different parameters, and the thickness was determined by ellipsometer. Using the conditions shown in Table 1, after sputtering for 5 min, the thicknesses of IGZO films and the deposition rate under different conditions were obtained as shown in Table 3. Next, see the corresponding experiment result analysis in Table 4 and Figure 1.

As can be seen from Table 4 and Figure 1, the deposition rate increased slightly from 24.4 to 27.2 nm/min at temperatures ranging from 100 to 180 °C, with a range of 2.8. For Ar/O_2_, the deposition rate of 30:3 was 36.2 nm/min, which is significantly higher than the 19.6 nm/min of 30:30 and extremely different to the rate for 16.6; in other words, the deposition rate tends to accelerate with the increase of the Ar/O_2_ ratio. When sputtering power increased from 80 to 140 W, the deposition rate increased from 18.6 to 33.4 nm/min, almost linearity, and the range was 14.8. According to the range analysis table and figures, the most important factor affecting the deposition rate of IGZO thin films at the current parameter levels is the Ar/O_2_ ratio, with the largest extreme difference of 16.6, followed by RF sputtering power and temperature. The deposition rate of IGZO film proposed was the fastest at 46.8 nm/min under C7 parameters.

The effects of sputtering parameters on the deposition rate can be explained as follows. At a high Ar/O_2_ ratio, enough Ar ions can reach the target surface and bombard target atoms. At this point, the sputtering atoms are less subject to secondary collisions and scattering, and can successfully fall on the substrate. As the ratio of O_2_ increases, the probability of sputtering atoms colliding and scattering increases, and the film deposition rate decreases. With the increase of sputtering power, the Ar ions get higher energy, and the more bombardment of the target, the higher the number of sputtered atoms, thereby increasing the deposition rate. The higher the temperature, the faster the rate of atomic thermal motion, and the quicker the sputtering atoms reach the base. However, it can be seen that temperature had little influence on the deposition rate of the films. This may have been due to the fact that temperature variations were small.

### 3.2. The Fabrication of a Crystalline IGZO Film

The sputtering time of C1–C9 was controlled by using the deposition rate results of Section 3.1 to obtain thin film samples with a thickness of 200 nm, labeled S1-1 to S9-1. The crystallographic quality of the samples was analyzed by XRD.

**Figure 2:**
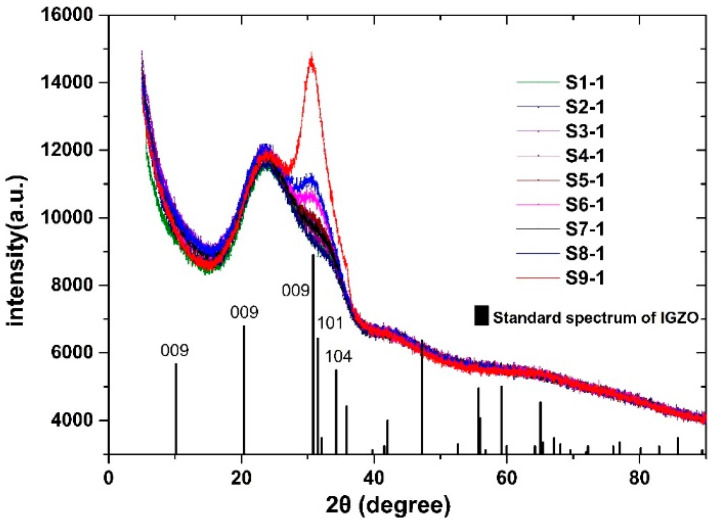
XRD spectrum of prepared IGZO thin films and the standard spectrum diagram of IGZO.

Figure 2 compares 2θ XRD spectra of IGZO films. The XRD images showed that all samples had a small hilly projection at about 25°, which is the diffraction peak of the glass substrate. With temperatures below 140 °C and an Ar/O_2_ ratio of 30:3, no samples showed crystallographic characteristics and the films prepared were a-IGZO. The S9-1 sample showed a significant diffraction peak around 31°; the S6-1 and S8-1 samples had a weak diffraction peak there, indicating crystal IGZO films were formed the S9-1 conditions [12]. According to the standard PDF database (PDF number 38-1104), the peak is derived from a (009) plane of crystal IGZO, indicating that IGZO film prepared by C9 had good c-axial crystallinity. Additionally, the interplanar spacing of S9-1 calculated from the XRD map is 0.292 nm. 

**Figure 3:**
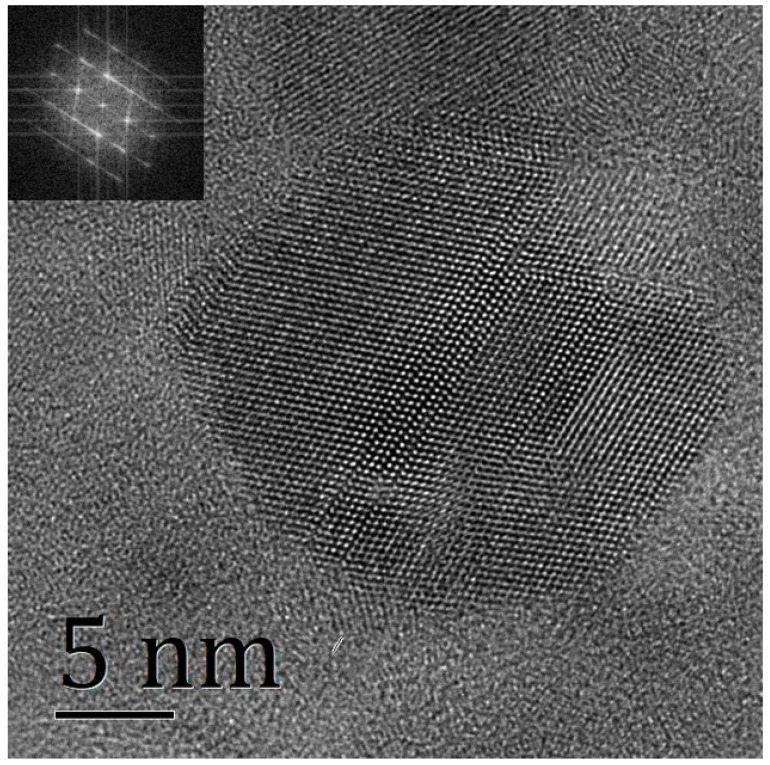
HRTEM image and FFT diffractogram (inset) of S9-1.

We further characterized the S9-1 thin films by HRTEM imaging. Figure 3 shows an HRTEM image of S9-1 powder on a copper wire screen, showing a clear crystal structure in the middle region. We used a Fast Fourier Transform (FFT) diffractogram (inset of Figure 3) of the HRTEM image to quantify the crystal structure of the prepared IGZO thin film and the expected hexagonal pattern appeared, indicating the IGZO thin film with a structure of c-axis aligned crystal.

**Figure 4:**
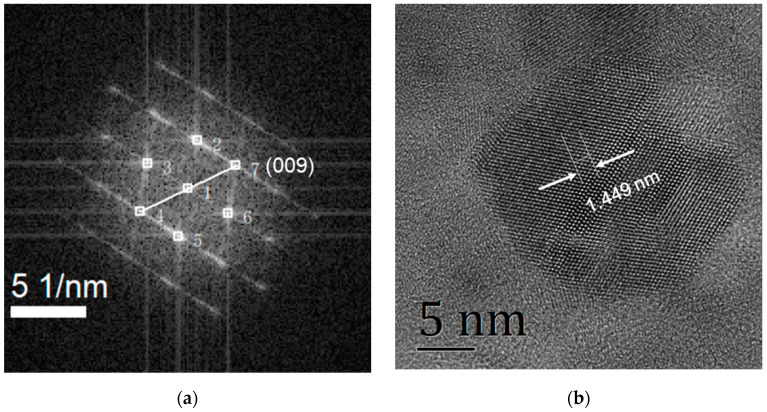
The interplanar spacing was obtained from the HRTEM image: (**a**) calibrated crystallographic orientation on an FFT diffractogram and (**b**) the corresponding interplanar spacing on an HRTEM image of five layers.

Moreover, we calibrated the grain orientation of the crystal IGZO by comparing the FFT diffractogram with the standard PDF card (PDF number 38-1104) and verified the 009 grain face, as shown in the Figure 4. The grain orientation was applied back to the HRTEM diagram for calculation of grain spacing. In accordance with the direction of the calibration in Figure 4a, we measured the grain surface distance of five layers on the HRTEM image. The measured value was 1.449 nm and the final interplanar spacing was 0.290 nm, which is basically consistent with the result calculated by using XRD map in Section 3.1. 

Based on the above analysis, we determined that crystallized IGZO films were generated by using C9. Comparison with other conditions, especially C6 and C8, led to the conclusion that the most important parameters affecting the crystallization state are temperature and O_2_ ratio. 

Many other oxide materials, such as ZnO, are known to form crystalline films at high temperatures [18,19]. The growth mechanism of sputtering crystal oxides includes nucleation on an initial disordered (about 5 nm) layer; subsequent ordered growth occurs if the substrate temperature is high enough to cause coalescence [20,21]. We believe that the crystallized IGZO film forms through a similar nucleation process in the early stages of deposition. At low temperatures, the mobility of the atoms is not enough to form these nuclei, or to grow laterally at a rate sufficient in order to coalesce into a coherent membrane. Above 140 °C, the films begin to grow more and more in an orderly fashion. The effect of the O_2_ ratio on the crystalline state is more pronounced at the stage of membrane growth. When the oxygen ratio is high, it is easier to form a complete crystal structure, and the probability of forming a disordered structure with more oxygen vacancy is reduced. While it is clear that nucleation of grains seems much easier with high O_2_ content, the direct connection between oxygen re-sputtering and alignment remains unclear.

### 3.3. The Corrosion Resistance of the Crystallized IGZO Film

**Figure 5:**
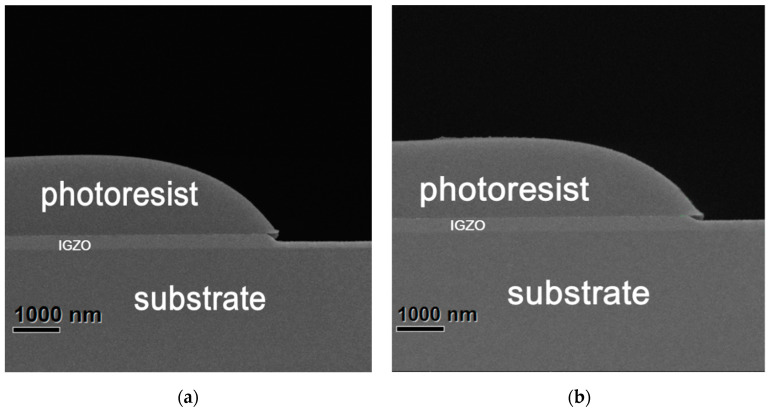
The cross-sectional SEM images of (**a**) S1-2 (a-IGZO film) and (**b**) S9-2 (crystallized IGZO film) after etching.

The crystallized IGZO film was expected to have better stability, and we compared its acid corrosion resistance with a-IGZO. IGZO thin film samples with 300 nm thickness were prepared on the glass substrate by using C1 and C9 from Table 3, labeled S1-2 (a-IGZO sample) and S9-2 (crystal IGZO sample) respectively. S1-2 and S9-2 were then coated with photoresists, and we exposed half of them after lithography development, and then they were placed in a dilute hydrochloric acid solution (HCL:H_2_O = 1:10 by volume) for 40 seconds each. Figure 5 shows the cross-sectional scanning electron microscope (SEM) diagrams of S1-2 and S9-2 after etching. According to the differential between the thickness of the unetched IGZO film under the protection of a photoresist and that of the unprotected film, the etching rate of two samples can be calculated. 

As can be seen from Figure 5, the etching depth of S1-2 was significantly greater than that of the S9-2, which means that a-IGZO thin film was more susceptible to acid corrosion than the proposed crystal IGZO film. We measured the height differences at the steps in Figure 5a,b to be 174.7 and 100.7 nm respectively. By dividing by time 40 s, we got the etching rates of 4.35 and 2.50 nm/s. This means that the corrosion resistance of the prepared crystalline IGZO is 74% higher than that of a-IGZO. The improvement may be due to a more compact structure of the crystalline IGZO.

## 4. Conclusions

In conclusion, we report the deposition of a crystallized IGZO thin film by RF sputtering. The effects of temperature, Ar/O_2_ ratio and power on film formation during RF sputtering were analyzed via DOE method. The experiment results show that only a-IGZO films can be obtained at low temperatures or for low O_2_ ratios. At 140 °C with a 33% O_2_ fraction, crystal formation begins and increases with increasing temperature and O_2_ ratio. At the current conditions, IGZO thin films with the best crystallization performance were obtained at 180 °C, O_2_ ratio 50% and power 110 W.

XRD images were used to characterize the structures of IGZO films. Film deposition under optimized conditions shows a significant diffraction peak around 31°, and the peak is derived from a (009) plane of crystal IGZO, indicating that the film has good crystallinity. The interplanar spacing calculated from XRD map was 0.292 nm. The crystallized film was further characterized by HRTEM. An HRTEM image and FFT diffractogram show that the prepared film had a clear crystal structure, and the interplanar spacing measured from the HRTEM image was 0.290 nm, which is basically consistent with the results calculated by using XRD.

The corrosion resistance of the prepared crystallized IGZO thin film was characterized by the etching rate. The experimental results show that the acid corrosion resistance of crystalline IGZO films can be increased by 74%, which indicates that this film can provide more stable performance in applications such as BCE TFTs.

The future work will focus on expanding sputtering parameters around C9 conditions, exploring the influences of sputtering parameters on the crystallinity of thin films and applying the prepared crystalline films to TFT devices to explore their effects on electrical properties.

## Figures and Tables

**Table 1:** Factors and levels used in RF sputtering.

Factors	Level 1	Level 2	Level 3
**Temperature (°C)**	100	140	180
**Ar/O_2_ (sccm/sccm)**	30:3	30:15	30:30
**Power (W)**	80	110	140

**Table 2:** Orthogonal table of RF sputtering parameters.

Condition	Temperature (°C)	Ar/O_2_ (sccm)	Power (W)
**1**	100	30:3	80
**2**	100	30:15	110
**3**	100	30:30	140
**4**	140	30:3	110
**5**	140	30:15	140
**6**	140	30:30	80
**7**	180	30:3	140
**8**	180	30:15	80
**9**	180	30:30	110

**Table 3:** Orthogonal table of IGZO film deposition rate.

Condition	Temp. (°C)	Ar/O_2_ (sccm)	Power (W)	THK. (nm)	D.R. (nm/min)
**1**	100	30:3	80	129	25.8
**2**	100	30:15	110	111	22.2
**3**	100	30:30	140	126	25.2
**4**	140	30:3	110	180	36
**5**	140	30:15	140	141	28.2
**6**	140	30:30	80	72	14.4
**7**	180	30:3	140	234	46.8
**8**	180	30:15	80	78	15.6
**9**	180	30:30	110	96	19.2

**Table 4:** Range analysis table of IGZO film deposition rate.

Averge data of D.R.	Temperature	Ar/O_2_	Power
**Level 1**	24.4	36.2	18.6
**Level 2**	26.2	22	25.8
**Level 3**	27.2	19.6	33.4
**Range**	2.8	16.6	14.8

## Data Availability

All data generated or analyzed during this study are included in this published article.
